# Gastric Balloon Complication Mimicking Acute Coronary Syndrome: A Case Report

**DOI:** 10.7759/cureus.96150

**Published:** 2025-11-05

**Authors:** Mosaab A Okache, Ibtihal E Mohamed, Alrand A Alakkasha, Halit Aldous

**Affiliations:** 1 Internal Medicine, Almana General Hospital, Dammam, SAU; 2 Emergency Medicine, Alsalkhadi Medical Center, Damascus, SYR; 3 Internal Medicine, Abant İzzet Baysal Üniversitesi Sağlık Araştırma Ve Uygulama Merkezi, Bolu, TUR

**Keywords:** acute kidney injury, bariatric endoscopy, gastric balloon, non-st-elevation myocardial infarction, troponin

## Abstract

Although intragastric balloons are a common endoscopic bariatric therapy, they carry a risk of complications, including obstruction and intolerance, which can lead to severe dehydration and metabolic disturbances. We present the case of a 40-year-old female with a history of ischemic cardiomyopathy and hypertension who presented to the emergency department with chest pain, abdominal pain, and repeated vomiting one month after gastric balloon insertion. Her initial workup was concerning for acute coronary syndrome, with EKG showing inferolateral ST-T depressions and a positive troponin. Further evaluation revealed severe acute kidney injury, metabolic alkalosis, and dehydration. The multidisciplinary team diagnosed sepsis and acute kidney injury due to severe dehydration from a suspected gastric outlet obstruction secondary to the balloon. Management involved fluid resuscitation, electrolyte correction, antibiotic therapy, and urgent endoscopic removal of the gastric balloon. Following removal, the patient’s symptoms, EKG changes, and laboratory abnormalities resolved completely. This case highlights a critical diagnostic pitfall: metabolic and renal sequelae of gastric balloon complications can mimic non-ST-elevation myocardial infarction, potentially leading to misdirected care. A high index of suspicion for non-cardiac causes is essential in patients with bariatric devices to ensure timely and correct intervention.

## Introduction

The intragastric balloon is a widely used endoscopic procedure for weight loss, offering a less invasive alternative to bariatric surgery. While generally safe, its known adverse effects include nausea, vomiting, abdominal pain, and gastroesophageal reflux. In a small but significant number of cases, more severe complications can occur, such as spontaneous hyperinflation, balloon migration, or gastric outlet obstruction [1]. These complications can lead to persistent vomiting, profound dehydration, and electrolyte imbalances.

Acute kidney injury (AKI) is a serious potential consequence of severe dehydration. Furthermore, the resulting metabolic disarray and systemic stress can cause secondary cardiac manifestations. Elevated troponin levels, a specific biomarker for myocardial injury, are not exclusively indicative of acute coronary syndrome. They can be elevated in various non-ischemic conditions, including sepsis, renal failure, and critical illness [2]. Similarly, electrolyte abnormalities, particularly hypokalemia, can precipitate significant EKG changes, including ST-segment and T-wave abnormalities, which can mimic ischemia [3].

We report a case where the complications of a gastric balloon, specifically, vomiting leading to dehydration, AKI, and metabolic alkalosis, presented with a clinical picture strongly mimicking a non-ST-elevation myocardial infarction (NSTEMI). This case underscores the importance of a broad differential diagnosis in patients with cardiac risk factors who present with chest pain and abnormal cardiac biomarkers, especially in the context of a recent medical device implantation.

## Case presentation

A 40-year-old female was brought to the emergency department via ambulance with acute-onset, severe epigastric and chest pain. The abdominal pain was described as heavy and burning, localized to the epigastrium, and was exacerbated by movement and breathing. It was associated with repeated vomiting, nausea, shortness of breath, left shoulder numbness, and abdominal bloating. The symptoms were not relieved by over-the-counter analgesics.

Her medical history was significant for hypertension and ischemic cardiomyopathy status post-percutaneous coronary intervention 12 years prior. Her home medications included aspirin, Entresto, Concor, rosuvastatin, esomeprazole, and empagliflozin. Notably, one month before presentation, she underwent insertion of a gastric balloon for weight loss. Following the procedure, she was advised to stop her antihypertensive medications. Over that month, she reported an inability to tolerate solid foods, consuming only light liquids, and had no bowel movements, with an associated 20 kg weight loss.

**Figure 1:**
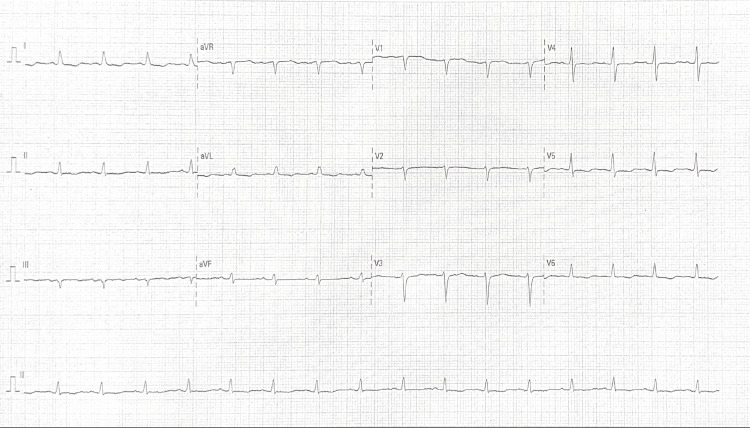
EKG showing inferolateral ST-T depressions.

On examination, the patient was in distress. Vital signs showed tachycardia (pulse of 120 beats/minute), tachypnea (respiratory rate of 30 breaths/minute), and hypertension (blood pressure of 140/90 mmHg). Oxygen saturation was 99% on room air. Abdominal examination revealed distension with rigidity and severe tenderness in the epigastric region. Bowel sounds were continuous. Chest examination was unremarkable. An EKG showed inferolateral ST-T depressions (Figure 1).

Initial laboratory investigations were significant for an elevated high-sensitivity troponin (0.054 ng/mL), AKI (creatinine of 468.8 U/L, blood urea nitrogen of 24.3 mg/dL), hypokalemia (3.3 mmol/L), and elevated hemoglobin (18.3 g/dL), suggestive of hemoconcentration. Venous blood gas revealed metabolic alkalosis (pH of 7.607, pCO_2_ of 28.0 mmHg, HCO_3_ of 26.4 mmol/L) (Table 1).

**Table 1:** Initial laboratory investigations.

Investigation	Result	Normal range
High-sensitivity troponin	0.054 ng/mL	<0.014 ng/mL
Creatinine	468.8 μmol/L	60–110 μmol/L
Blood urea nitrogen	24.3 mg/dL	7–20 mg/dL
Potassium	3.3 mmol/L	3.5–5.0 mmol/L
Hemoglobin	18.3 g/dL	13.0–17.0 g/dL
pH	7.607	7.32–7.43
PCO_2_	28.0 mmHg	38–50 mmHg
HCO_3^-^_	26.4 mmol/L	22–26 mmol/L

**Figure 2:**
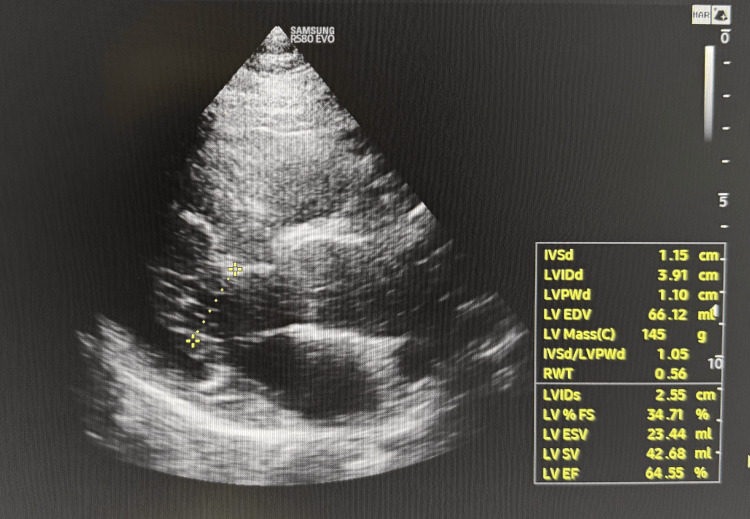
Transthoracic echocardiogram showing mild concentric left ventricular hypertrophy.

A transthoracic echocardiogram showed mild concentric left ventricular hypertrophy but no new regional wall motion abnormalities (Figure 2).

**Figure 3:**
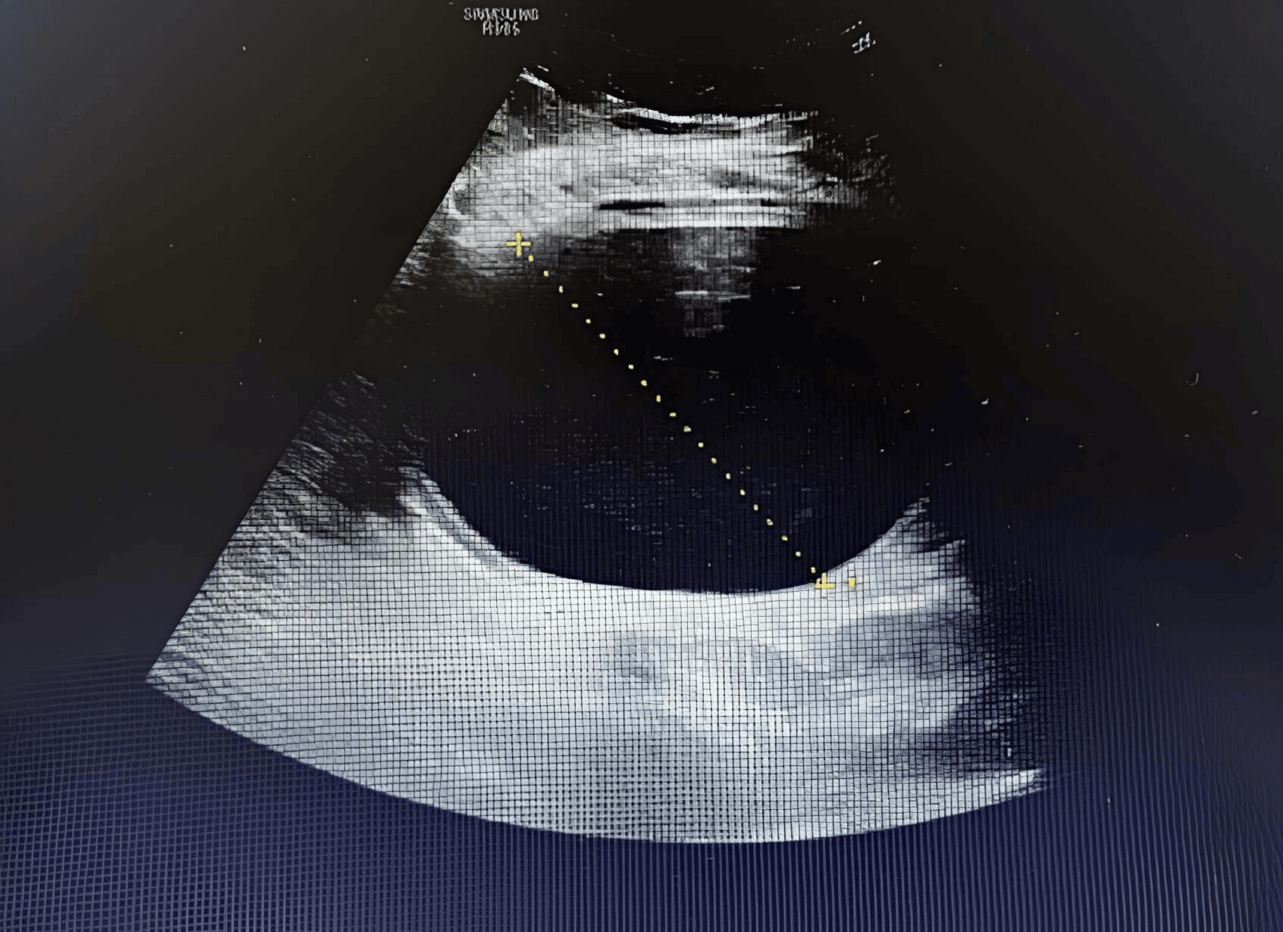
Abdominal ultrasound showing a fluid-filled gastric balloon in the antrum.

An abdominal ultrasound confirmed the presence of a fluid-filled gastric balloon in the antrum (Figure 3).

The patient was admitted to the intensive care unit (ICU). A multidisciplinary team involving ICU, cardiology, gastroenterology, and surgery concluded that the primary issues were sepsis, severe dehydration, and AKI secondary to a possible gastric outlet obstruction from the balloon, leading to metabolic alkalosis from persistent vomiting. The cardiac enzymes and EKG changes were attributed to a type 2 myocardial infarction in the setting of this systemic stress and electrolyte imbalance.

The management plan was comprehensive. The patient was kept nil per os (NPO) and started on aggressive intravenous fluid resuscitation with dextrose 25% and potassium repletion. A sepsis bundle was initiated, including empiric antibiotics (Tazocin) at a renally adjusted dose. The definitive intervention was an urgent upper gastrointestinal endoscopy, which was performed successfully to remove the gastric balloon.

The endoscopic procedure was performed successfully, and the gastric balloon was removed without complication. Post-removal, the patient’s clinical condition improved dramatically. Her abdominal pain resolved, and she was gradually advanced to an oral diet. Her renal function, electrolyte levels, and EKG normalized over the following days. She was discharged in stable condition with instructions to follow up in the outpatient clinic and resume her cardiac medications.

## Discussion

This case illustrates a complex presentation where a complication of a non-cardiac device mimicked acute coronary syndrome. The patient’s known cardiac history rightly raised alarm for an NSTEMI upon arrival. However, the root cause was a severe metabolic derangement stemming from gastric balloon intolerance.

The postulated sequence of events was as follows: the gastric balloon likely caused a functional gastric outlet obstruction or severe gastroparesis, leading to persistent vomiting. This resulted in three key interrelated problems. First, severe dehydration and pre-renal AKI: the inability to intake fluids and excessive gastric losses led to volume depletion, evidenced by tachycardia, elevated hemoglobin, and a sharp rise in creatinine. Pre-renal AKI is a well-documented complication of intractable vomiting [4]. Second, metabolic alkalosis: the loss of gastric acid (HCl) from vomiting leads to a net gain of bicarbonate in the blood, causing metabolic alkalosis, which was clearly demonstrated in this patient’s blood gas results [5]. Third, electrolyte imbalances: vomiting causes loss of potassium and hydrogen ions. The resulting hypokalemia can directly affect cardiac myocyte repolarization, leading to EKG changes such as ST-segment depression and T-wave flattening, which can be mistaken for myocardial ischemia [3].

The elevated troponin in this context is consistent with a type 2 myocardial infarction, where a demand-supply mismatch occurs not from coronary plaque rupture, but from another stressor; in this case, sepsis, hypotension, and tachycardia in a patient with underlying cardiomyopathy [2]. The combination of AKI and sepsis further contributes to troponin elevation through reduced clearance and systemic inflammation [6].

This case aligns with the literature on the complications of intragastric balloons. A systematic review by Abu Dayyeh et al. found that early intolerance occurs in approximately 7% of cases, sometimes requiring early removal [1]. While severe AKI requiring ICU admission is less commonly reported, it represents a critical, life-threatening chain of events that clinicians must recognize.

The key learning point is the necessity of a holistic diagnostic approach. In a patient with a cardiac history, it is crucial not to anchor prematurely on a cardiac diagnosis when a clear, non-cardiac precipitant is present. The absence of new wall motion abnormalities on echocardiogram and the rapid resolution of both EKG changes and troponin elevation after correcting the underlying metabolic problem strongly support the conclusion that the primary event was not an acute coronary occlusion.

## Conclusions

This case illustrates a critical clinical scenario where a severe metabolic and renal complication from a gastric balloon mimicked acute coronary syndrome. The patient presented with a clinical picture highly suggestive of an NSTEMI, including chest pain, characteristic EKG changes, and elevated troponin levels. However, the root cause was not a primary cardiac event but rather a cascade of events originating from gastric balloon intolerance, which led to persistent vomiting, profound dehydration, and AKI. The resulting electrolyte imbalances and systemic stress created a perfect storm that manifested with cardiac biomarkers and EKG findings indistinguishable from ischemia. The rapid resolution of all symptoms and normalization of all laboratory parameters following balloon removal and supportive care confirm this diagnosis. This report underscores the importance of maintaining a broad differential diagnosis, especially in patients with cardiac risk factors who present with symptoms shortly after a medical device implantation. Clinicians must be aware that severe complications from bariatric interventions can have systemic, life-threatening consequences that masquerade as other conditions. A high index of suspicion for such mimics is essential to avoid misdirected treatment and to ensure timely, appropriate intervention that addresses the underlying cause.
